# Enthalpies of Combustion and Formation of Severely Crowded Methyl-Substituted 1,3-dioxanes. The Magnitudes of 2,4- and 4,6-diaxial Me,Me-Interactions and the Chair–2,5-twist Energy Difference

**DOI:** 10.3390/molecules25122762

**Published:** 2020-06-15

**Authors:** Kalevi Pihlaja, Henri Kivelä, Pirjo Vainiotalo, William V. Steele

**Affiliations:** 1 Department of Chemistry, University of Turku, FI-20500 Turku, Finland; 2 Department of Chemistry, University of Joensuu, FI-80100 Joensuu, Finland; pirjo.vainiotalo@joensuu.fi; 3 Department of Chemistry, University of Stirling, Stirling FK9 4LA, Scotland, UK; wsteele@utk.edu

**Keywords:** crowded 1,3-dioxanes, enthalpies of combustion, enthalpies of formation, chair-2,5-twist energy difference, syn-axial Me,Me-interactions

## Abstract

Enthalpies of combustion of 2,2-*trans*-4,6- (**1**) and 4,4,6,6-tetramethyl- (**2**) and 2,4,4,6,6- (**3**) and 2,2,4,4,6-pentamethyl-1,3-dioxanes (**4**) were determined to estimate their enthalpies of formation in the gas phase. By comparing the latter with the corresponding enthalpies estimated based on the various bond–bond interactions allowed to determine the chair–2,5-twist energy difference (Δ*H*_CT_ = 29.8 kJ mol^–1^) for **1** since C-13 shift correlations indicate that it escapes to the 2,5-twist form where the 2-methyl groups are isoclinal and 4- and 6-methyl groups pseudoequatorial to avoid syn-axial interactions. Compounds **2** and **3** in turn give the values 21.0 and 21.6 kJ mol^–1^ for the 4,6-diaxial Me,Me-interaction. Finally compound **4**, which retains the chair conformation to avoid pseudoaxial interactions in the twist forms gives the value 19.5 kJ mol^–1^ for the 2,4-diaxial Me,Me-interaction indicating that its chair form appears to be somewhat deformed.

## 1. Introduction

We have thoroughly studied the conformations of various heterocycles [1], especially those of methyl-substituted 1,3-dioxanes [1] (pp. 91–96). Our oxygen-containing compounds allowed us, together with the literature data [2], to assemble sets of bond–bond interactions which together with the enthalpies of formation of gaseous atoms and bond energies allow the estimation of enthalpies of formation. The latter do not cover e.g., the 2,4- and 4,6-diaxial Me,Me-interactions present in 2,2,4,4,6-pentamethyl- (**4**) or 4,4,6,6-tetra- (**2**) and 2,4,4,6,6- pentamethyl-1,3-dioxanes (**3**) and e.g., the chair–2,5-twist energy difference in 1,3-dioxanes. This is why we have determined earlier the enthalpies of combustion and formation of gaseous 2,2-*trans*-4,6-tetramethyl-1,3-dioxane [3,4] (**1**) which is known, based on C-13 chemical shift correlations, to attain a 2,5-twist form [1,5] and determine those of **2**–**4** which, again based on C-13 chemical shift correlations [1,5], seem to retain the chair conformation [1,4].

## 2. Results

### 2.1. Preparation of the Studied Compounds

#### 2.1.1. Starting Materials

- 2-Methyl-2,4-pentanediol was a commercial product from Fluka AG (Buchs, Switzerland).
- 2,4-Dimethyl-2,4-pentanediol was prepared with Grignard reaction from ethyl β-methyl,β- hydroxybutyrate and methyliodide [6]. Its boiling point was 363–365 K at 1.7 kPa.

#### 2.1.2. 1,3-Dioxanes

**Scheme 1:**
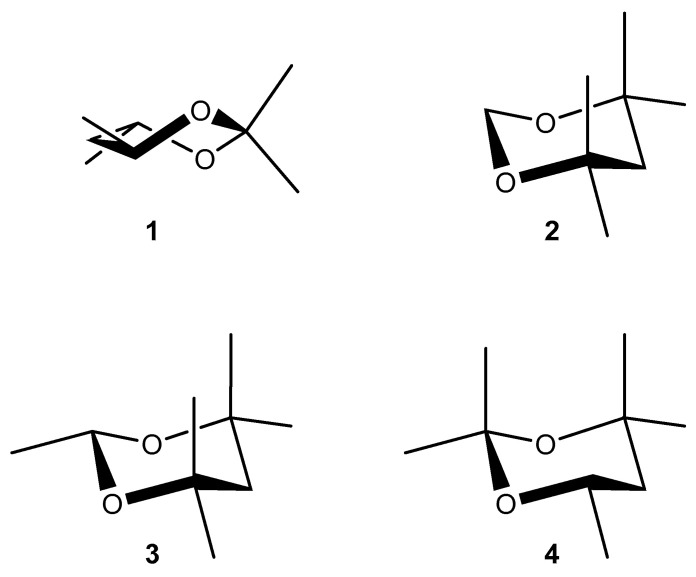
Predominant conformations of **1**–**4**. **1**: 2,2-*trans*-4,6-dimethyl-1,3-dioxane (4,6-dipseudo- equatorial 2,5-twist), **2**: 4,4,6,6-tetramethyl-1,3-dioxane (chair), **3**: 2,4,4,6,6-pentamethyl-1,3-dioxane (2-equatorial chair), **4**: 2,2,4,4,6-pentamethyl-1,3-dioxane (6-equatorial chair) [12].

- 4,4,6,6-tetramethyl-1,3-dioxane (**2**) was prepared with the method developed by Rondestvedt [7] from 2,4-dimethyl-2,4-pentanediol and paraformaldehyde (formaldehyde polymer) in dichloromethane using *p*-toluenesulfonic acid as catalyst. After the distillation, the product was allowed to stand on saturated sodium bisulfite solution until all unreacted aldehyde was removed. Final purification was carried out on a Perkin Elmer F 21 preparative gas chromatograph using a 4.5 m steel column including 20% Carbowax 20M as the liquid phase and Chromosorb G (60/80 mesh) as the solid phase. The boiling point was 349 K at 6.9 kPa and 427.6 K at normal pressure. Water content was 0.05 ± 0.005% (Scheme 1).
- 2,4,4,6,6-pentamethyl-1,3-dioxane (**3**) was prepared by boiling equimolar amounts of paraldehyde (acetaldehyde polymer) and 2,4-dimethyl-2,4-pentanediol in hexane with *p*-toluenesulfonic acid as catalyst in a water entrainment unit [7]. The raw product was purified as above. The boiling point was 353 K at 8.6 kPa and 423.7 K at normal pressure. Water content was 0.06 ± 0.005%.
- 2,2,4,4,6-pentamethyl-1,3-dioxane (**4**) was prepared by boiling equimolar amounts of acetone and 2-methyl-2,4-pentanediol in hexane with *p*-toluenesulfonic acid as catalyst in a water entrainment unit [7]. The raw product was purified as above. The boiling point was 420.1 K at normal pressure. No water was found. For NMR characterization of **1**–**4**, see Refs. [5,8,9,10,11].

### 2.2. Combustion Experiments

The enthalpies of combustion and formation of gaseous *trans*-2,2,4,6-tetramethyl-1,3-dioxane (**1**) were published earlier [3,4]. Those of compounds **2**–**4** were determined as described in Materials and Methods and are listed in Table 1, Table 2 and Table 3. Table 4 in turn lists the enthalpies of formation of gaseous atoms, the bond energies and the bond–bond interactions [2] for the estimation of the enthalpies of formation of theoretically strain free gaseous compounds **1** to **4** shown in Scheme 1.

## 3. Discussion

The enthalpies of vaporization of gaseous **1**–**4** (Scheme 1) were estimated from Equation (1) which Wadsö derived for weakly associated compounds [12]. E.g., in the case of nine gaseous secondary amines [13] the calculated enthalpies of vaporization deviated on the average from the experimental ones only by ± 0.5 kJ mol^−1^.

(1) $$\Delta H_{\mathrm{vap}}(25{}^{\circ}C)/{\mathrm{kJ}\;\mathrm{mol}}^{-1}=20.9+0.172\left(t_{\mathrm{bp}}/{}^{\circ}C\right).$$

Compound **1**: The theoretical strain-free (sf) enthalpy of formation for gaseous **1** was obtained from the following equation (the various parameters are given in Table 4):

(2) $$\begin{array}{l} -\Delta H_{f,\mathrm{sf}}{}^{\circ}\left(1,g\right)=-8\Delta H_{f}{}^{\circ}\left(C,g\right)-16\Delta H_{f}{}^{\circ}\left(H,g\right)-2\Delta H_{f}{}^{\circ}\left(O,g\right)+6E_{b}\left(\mathrm{CC}\right)+16E_{b}\left(\mathrm{CH}\right)+4E_{b}\left(\mathrm{CO}\right)\\ +4\Gamma_{\mathrm{CCC}}+8\Gamma_{\mathrm{CCO}}+2\Gamma_{\mathrm{COC}}+\Gamma_{\mathrm{OCO}}+4\Delta_{\mathrm{CCO}}+2\Delta_{\mathrm{OCO}}\\ =-5733.6-3488.0-498.4+1980.6+6653.6+1311.8+46.8+192.08+47.44+54.75–26.08–28.6\\ =512.5\;\mathrm{kJ}\;{\mathrm{mol}}^{-1}. \end{array}$$

The corrected experimental enthalpy of formation of liquid **1**, −526.3 kJ mol^−1^, was given in Ref. [4]. The enthalpy of vaporization of **1** evaluated from equation (1) (*t*_bp_ = 132.2 °C) equals 43.6 kJ mol^−1^ and hence Δ*H*_f_°(**1**,g) = −482.7 kJ mol^−1^. Accordingly the value of Δ*H*_CT_(2,5) equals 512.5−482.7 = 29.8 kJ mol^−1^ which was already quoted in Ref. [5].

Compounds **2** and **3**:

(3) $$\begin{array}{l} -\Delta H_{f,\mathrm{sf}}{}^{\circ}\left(2,g\right)=-8\Delta H_{f}{}^{\circ}\left(C,g\right)-16\Delta H_{f}{}^{\circ}\left(H,g\right)-2\Delta H_{f}{}^{\circ}\left(O,g\right)+6E_{b}\left(\mathrm{CC}\right)+16E_{b}\left(\mathrm{CH}\right)+4E_{b}\left(\mathrm{CO}\right)\\ +7\Gamma_{\mathrm{CCC}}+6\Gamma_{\mathrm{CCO}}+2\Gamma_{\mathrm{COC}}+\Gamma_{\mathrm{OCO}}+2\Delta_{\mathrm{CCC}}+6\Delta_{\mathrm{CCO}}\\ =-5733.6-3488.0-498.4+1980.6+6653.6+1311.8+82.0+144.07+47.44+54.75-6.54-39.12\\ =508.6\;\mathrm{kJ}\;{\mathrm{mol}}^{-1}. \end{array}$$

The experimental enthalpy of formation of gaseous **2**, −470.6 kJ mol^−1^, is given in Table 1. Thus, the total strain in **2** is 508.6–470.6 = 38.0 kJ mol^−1^. This is including two 2-H_ax_,4-Me_ax_ interactions [4] (17.0 kJ mol^−1^) together with the syn-axial 4,6-Me,Me interaction. Accordingly, the latter is equal to 38.0−17.0 = 21.0 kJ mol^−1^.

Similarly:

(4) $$\begin{array}{l} -\Delta H_{f,\mathrm{sf}}{}^{\circ}\left(3,g\right)=-9\Delta H_{f}{}^{\circ}\left(C,g\right)-18\Delta H_{f}{}^{\circ}\left(H,g\right)-2\Delta H_{f}{}^{\circ}\left(O,g\right)+7E_{b}\left(\mathrm{CC}\right)+18E_{b}\left(\mathrm{CH}\right)+4E_{b}\left(\mathrm{CO}\right)\\ +7\Gamma_{\mathrm{CCC}}+8\Gamma_{\mathrm{CCO}}+2\Gamma_{\mathrm{COC}}+\Gamma_{\mathrm{OCO}}+2\Delta_{\mathrm{CCC}}+6\Delta_{\mathrm{CCO}}+\Delta_{\mathrm{OCO}}\\ =-6450.3-3924.0-498.4+2310.7+7485.3+1311.8+82.0+192.08+47.44+54.75-6.54-39.12-14.3\\ =551.4\;\mathrm{kJ}\;{\mathrm{mol}}^{-1}. \end{array}$$

The evaluated enthalpy of gaseous **3**, −512.8 kJ mol^−1^, is given in Table 2. Thus the total strain in **3** is 551.4 − 512.8 = 38.6 kJ mol^−1^. This includes again two 2-H_ax_,4-Me_ax_ interactions [4] (17.0 kJ mol^−1^) together with the syn-axial 4,6-Me,Me interaction. Accordingly, the latter equals 38.6 − 17.0 = 21.6 kJ mol^−1^. So on the average 4,6-diaxial Me,Me-interaction equals 21.3 kJ mol^−1^.

In order to determine syn-axial 2,4-Me,Me-interaction we must evaluate the theoretical enthalpy of formation for the strain-free gaseous **4**:

(5) $$\begin{array}{l} -\Delta H_{f,\mathrm{sf}}{}^{\circ}\left(4,g\right)=-9\Delta H_{f}{}^{\circ}\left(C,g\right)-18\Delta H_{f}{}^{\circ}\left(H,g\right)-2\Delta H_{f}{}^{\circ}\left(O,g\right)+7E_{b}\left(\mathrm{CC}\right)+18E_{b}\left(\mathrm{CH}\right)+4E_{b}\left(\mathrm{CO}\right)\\ +6\Gamma_{\mathrm{CCC}}+9\Gamma_{\mathrm{CCO}}+2\Gamma_{\mathrm{COC}}+\Gamma_{\mathrm{OCO}}+\Delta_{\mathrm{CCC}}+6\Delta_{\mathrm{CCO}}+2\Delta_{\mathrm{OCO}}\\ =-6450.3-3924.0-498.4+2310.7+7485.3+1311.8+70.3+216.1+47.44+54.75-3.27-39.12-28.6\\ =552.6\;\mathrm{kJ}\;{\mathrm{mol}}^{-1}. \end{array}$$

The experimental enthalpy of formation of gaseous **4**, −520.9 kJ mol^−1^, is given in Table 3. Accordingly, the total interactions in **4** are 552.6 − 520.9 = 31.7 kJ mol^−1^. This includes one 2-Me_ax_,6-H_ax_ [4], one 4-Me_ax_,6-H_ax_ interaction [4], i.e., 8.5 + 3.7 = 12.2 kJ mol^−1^. Thus the magnitude of 2,4-diaxial Me,Me-interaction is equal to 31.7 − 12.2 = 19.5 kJ mol^−1^. In other words the syn-axial Me,Me-interactions do not differ too much from each other but appear to indicate that the chair form of **4** can be somewhat more deformed than those of **2** and **3**. If compound **1** would exist also in the chair form its total strain should be practically equal to that, 31.7 kJ mol^−1^, in **4**. However, it is only 29.8 kJ mol^−1^ which supports its existence in the 2,5-twist form.

## 4. Materials and Methods

The standard enthalpy of combustion of 2,2,4,4,6-pentamethyl-1,3-dioxane (**4**) was determined on the high-precision aneroid static-bomb combustion calorimeter built and tested in Stirling. The detailed structure and procedure were as described earlier [14]. The standard enthalpies of combustion of 4,4,6,6-tetra- (**2**) and 2,4,4,6,6-pentamethyl-1,3-dioxanes (**3**) were in turn determined by burning them in oxygen in an adiabatic bomb calorimeter No. 1221 manufactured by Parr instruments Co., Illinois, USA. The bomb and procedure were described earlier as well [3,4].

## Figures and Tables

**Table 1:** Combustion results of 4,4,6,6-tetramethyl-1,3-dioxane (**2**, C_8_H_16_O_2_). *e*°(calor.) = (10219.8 ± 2.5) J K^−1^, −Δ*u*_c_°(gel.) = (18817.3 ± 8.4) J g^−1^, Δ*nRT* = −7.4 kJ mol^−1^.

			Experiments ^1^					
	1.	2.	3.	4.	5.	6.	7.	8.
*m*(**2**)/g	0.51757	0.49777	0.47285	0.53899	0.47061	0.54141	0.55610	0.53846
*m*(gel.)/g	0.12393	0.12088	0.12393	0.12193	0.12535	0.12237	0.12395	0.12592
*e*^f^(cont.)/ JK^−1^	17.77	17.62	17.55	17.85	17.55	17.85	17.92	17.85
Δ*T*/K	1.9594_4_	1.8872_2_	1.8122_2_	2.0288_8_	1.8066_6_	2.0372_2_	2.0900_0_	2.0344_4_
*q*(ign)/J	67.78	37.32	67.36	68.53	65.14	82.26	71.80	83.81
*q*(HNO_3_)/J	32.30	40.58	40.00	43.30	38.83	44.22	44.94	43.47
*q*(H_2_SO_4_)/J	4.06	3.97	4.06	4.02	4.14	4.02	4.06	4.14
*q*(gel.)/J	2332.04	2274.63	2332.04	2294.38	2358.73	2302.66	2332.41	2369.48
*q*_∑_/J	7.49	7.20	6.86	7.78	6.82	7.82	8.03	7.78
−Δ*u*_c_°/ kJg^−1^	34.0366	34.0652	34.0532	34.0508	34.0448	34.0138	34.0509	34.0216
−Δ*U*_c_°/ kJmol^−1^	4908.5	4912.6	4910.9	4910.6	4909.7	4905.2	4910.6	4906.4
			**Results (kJ mol^−1^)** ^2^					
Δ*U*_c_°(l)	−4909.3 ± 2.4		Δ*H*_vap_	47.4 ± 2				
Δ*H*_c_°(l)	−4916.7 ± 2.4		Δ*H*_f_°(g)	−470.6 ± 4.1				
Δ*H*_f_°(l)	−518.0 ± 3.2							

^1^ For explanation of the quantities, see Refs. [3,4,13]. ^2^ The values refer to 298.15 K.

**Table 2:** Combustion results of 2,4,4,6,6-pentamethyl-1,3-dioxane (**3**, C_9_H_18_O_2_). *e*°(calor.) = (10215.7 ± 2.1) J K^−1^, −Δ*u*_c_°(gel.) = (19440.2 ± 6.4) J g^−1^, Δ*nRT* = −8.7 kJ mol^−1^.

				Experiments ^1^							
	1.	2.	3.	4.	5.	6.	7.	8.	9.	10.	11.
*m*(**3**)/mg	459.317	454.329	429.039	471.094	510.543	514.600	468.487	461.188	454.690	434.877	488.729
*m*(gel)/mg	126.090	133.500	122.400	123.635	121.10	123.330	115.245	126.145	128.000	130.035	128.375
*e*^f^(cont.)/JK^−1^	17.55	17.55	17.40	17.62	17.77	17.77	17.55	17.55	17.55	17.47	17.70
Δ*T*/K	1.82555	1.82000	1.71166	1.85944	1.98722	2.00944	1.83444	1.82888	1.81166	1.74777	1.92611
*q*(ign)/J	63.86	61.50	66.19	73.81	40.42	62.68	56.82	59.75	61.50	65.02	41.00
*q*(HNO_3_)/J	39.37	36.69	35.56	39.83	41.55	41.97	38.66	39.37	38.07	40.17	41.21
*q*(gel.)/J	2451.24	2595.29	2379.48	2403.50	2354.42	2397.56	2240.41	2452.28	2488.35	2451.24	2595.29
*q*_∑_/J	6.69	6.61	6.23	6.86	7.45	7.49	6.82	6.69	6.61	6.32	7.11
−Δ*u*_c_°/ kJg^−1^	35.0883	35.0407	35.0182	35.0253	35.0755	35.0379	35.0614	35.0253	35.0574	35.0481	35.0347
−Δ*U*_c_°/kJmol^−1^	5552.4	5544.8	5541.3	5542.4	5544.4	5550.3	5548.1	5542.4	5547.5	5546.0	5543.9
			**Results (kJ mol^−1^)** ^2^								
Δ*U*_c_°(l)	−5545.8 ± 2.8			Δ*H*_vap_		46.8 ± 2					
Δ*H*_c_°(l)	−5554.5 ± 2.8			Δ*H*_f_°(g)		−512.8 ± 5.8					
Δ*H*_f_°(l)	−559.6 ± 3.8										

^1^ For explanation of the quantities, see Refs. [3,4,13]. ^2^ The values refer to 298.15 K.

**Table 3:** Results of the combustions of 2,2,4,4,6-pentamethyl-1,3-dioxane (**4**, C_9_H_18_O_2_). *ρ*(**4**) = 0.9240 g cm^−3^, *ε*(calor.) = 2261.6 ± 0.7 J Ω^−1^, *c*_p_(**4**) = 2.07 J K^−1^ g^−1^, Δ*u*_c_°(cotton fuse) = −16.240 kJ g^−1^,Δ*u*_c_°(polythene) = (−46.350 ± 0.015) kJ g^−1^, Δ*nRT* = −11.15 kJ mol^−1^.

		Experiments ^1^			
	1.	2.	3.	4.	5.
*m*(**4**)/g	0.025647	0.027543	0.035429	0.027748	0.025409
*m*(polythene)/g	0.020400	0.020750	0.021006	0.020076	0.021756
Δ*m*(cotton)/g	0.001375	0.001198	0.001354	0.001452	0.001259
*n*^l^(H_2_O)/mol	0.05551	0.05551	0.05551	0.05551	0.05551
Δ*R*/Ω	0.821414	0.857100	0.984095	0.847644	0.844729
−Δ*R*ε(calor.)/kJ	1.85771	1.93842	2.22563	1.91658	1.91044
−Δ*T*ε(cont.)/kJ	0.01257	0.01312	0.01506	0.01296	0.01293
Δ*E*(Wash.)/kJ	0.00350	0.00370	0.00484	0.00378	0.00348
Δ*E*(ign)/kJ	0.00178	0.00125	0.00118	0.00100	0.00145
−Δ*u*_c_°/kJg^−1^	34.9811	34.9886	34.9530	34.9813	35.0112
		**Results (kJ mol^−1^)** ^2^			
Δ*u*_c_°(l)	(−34.9830 ± 0.018) kJ g^−1^		Δ*H*_f_°(l)	−567.1	
Δ*U*_c_°(l)	−5535.85 ± 2.94		Δ*H*_vap_	46.2	
Δ*H*_c_°(l)	−5547.00 ± 2.94		Δ*H*_f_°(g)	−520.9	

^1^ For explanation of the quantities, see Ref. [14]. ^2^ The values refer to 298.15 K.

**Table 4:** The values of the enthalpies of formation Δ*H*_f_° of gaseous atoms, the bond energies *E*_b_ and the bond–bond interactions needed to estimate the enthalpies of formation for the theoretically strain-free gaseous compounds **1** to **4**.

Δ*H*_f_°/kJ mol^−1^		*E*_b_/kJ mol^−1^		Bond–Bond Interactions/kJ mol^−1^	
C(g)	716.7	C–C	330.1	Γ_CCC_	11.71
H(g)	218.0	C–H	415.85	Γ_CCO_	24.01
O(g)	249.2	C–O	327.95	Γ_COC_	23.72
				Γ_OCO_	54.75
				Δ_CCC_	−3.27
				Δ_CCO_	−6.52
				Δ_OCO_	−14.30
